# Disparities in fatigue levels and dietary habits between men and women with inflammatory bowel disease: a comparative analysis with a control cohort

**DOI:** 10.1007/s00394-026-03902-2

**Published:** 2026-02-16

**Authors:** Lea Pueschel, Heiner Wedemeyer, Henrike Lenzen, Miriam Wiestler

**Affiliations:** 1 Department of Gastroenterology, Hepatology, Infectious Diseases and Endocrinology, Hannover Medical School, 30625 Hannover, Germany; 2 Department of Gastroenterology, Hepatology, Interventional Endoscopy and Diabetology, Academic Teaching Hospital Braunschweig, 38126 Braunschweig, Germany; 3 PRACTIS Clinician Scientist Program, Dean’s Office for Academic Career Development, Hannover Medical School, Hannover, Germany

**Keywords:** Fatigue, Inflammatory bowel disease, Sex-differences, Diet quality

## Abstract

**Purpose:**

There is a paucity of research on sex differences in inflammatory bowel disease (IBD), particularly in relation to diet and fatigue.

**Methods:**

This is a subanalysis of a monocentric cross-sectional study that was performed at a tertiary referral center, with the study population comprising individuals diagnosed with IBD (*n* = 233) and a control cohort (CC; *n* = 96).

**Results:**

The mean FACIT sum scores were found to be significantly lower for individuals with IBD compared to CC (*p* = 0.005; g = 0.3). Additionally, women with IBD had significantly lower scores compared to men with IBD (25 to 29; *p* = 0.009; g = − 0.3). This trend was further supported by the observation of stronger declines in quality of life and food-related quality of life in women with IBD (*p* < 0.001; g = − 0.8; and *p* = 0.004; g = 0.6). Additionally, objective parameters of IBD activity (fecal calprotectin) and inflammation (C-reactive protein [CRP]) exhibited significant differences between fatigue levels in women with IBD (*p* = 0.003; g = − 0.4; and *p* = 0.034; g = − 0.3). The comparative cohort analysis revealed a prevailing trend of suboptimal dietary habits among men with IBD and women, both with and without fatigue, when contrasted with the control cohort. Detailed analysis of dietary patterns in relation to fatigue revealed discernible trends especially in men with IBD indicating a more adaptive pattern.

**Conclusion:**

This thorough analysis sheds further light on the complex interplay between dietary habits, psychosocial factors and fatigue in individuals with IBD, with a particular focus on sex-specific aspects.

**Supplementary Information:**

The online version contains supplementary material available at 10.1007/s00394-026-03902-2.

## Introduction

For individuals diagnosed with inflammatory bowel disease (IBD), which includes both Crohn’s disease (CD) and ulcerative colitis (UC), fatigue represents a pervasive and substantial complaint [1]. The hallmark characteristics of fatigue include extreme tiredness and a marked absence of energy, with the result that daily activities are significantly impeded. Notably, fatigue does not subside with rest, in contrast to the way tiredness does [2]. Consequently, fatigue exerts a pervasive effect on cognitive and physical performance - with nutritional deficiencies identified as a salient factor contributing to fatigue, particularly among elderly individuals and those afflicted with chronic conditions [3]. Insufficient nutrition and malnutrition have been demonstrated to be associated with fatigue, as inadequate protein and energy intake can result in the catabolism of body stores, leading to the depletion of fat and muscle. Moreover, deficiencies in vitamins and minerals have been associated with fatigue [3–5]. Conversely, overnutrition, characterized by the consumption of excessive amounts of high-fat and high-sugar diets, may also result in the occurrence of subjective fatigue symptoms [3]. Further findings show that a disturbance in metabolic homeostasis - as has been documented among diverse populations - contributes to fatigue [6–8]. Meanwhile, studies have demonstrated an association of inflammation and fatigue [9–12], with approximately 72% of individuals with IBD with active disease experiencing fatigue. However, the observation that nearly half of the patients in remission (47%) also reported experiencing fatigue suggests the potential involvement of additional mechanisms [13]. A thorough review of the existing literature indicates an intricate and multifaceted relationship between fatigue and IBD; yet there is a paucity of information regarding the underlying mechanisms and implications of the influence of nutrition. In addition, a growing body of research has begun to unravel the complex interplay between sex, fatigue, and its associated symptoms. Studies have demonstrated that fatigue is susceptible to bias related to gender/sex [14], and it is recognized that experiences of fatigue vary considerably between men and women. Indeed, there is a preponderance of research not only indicating that Chronic Fatigue Syndrome (CFS) affects a higher proportion of women [15], but also that overall women show higher levels of fatigue [16, 17]. However, the relationship between sex and fatigue varies across diverse fatigue types, such as cognitive fatigue [16]. Nevertheless, research in this domain still remains limited [18], yet the existing studies suggest the presence of sex-related differences in fatigue experiences, with implications for the diagnosis, treatment, and management of this condition. This lack of research is particularly pronounced in the context of sex disparities in the fatigue experience of individuals with IBD, underscoring the need for further investigation. Thus, the objective of this study was to provide additional insights into the multifaceted relationship between dietary habits and fatigue in patients with IBD, with a sex-specific focus and in comparison, to a control cohort.

## Materials and methods

The current subanalysis constitutes a segment of a broader investigative endeavor, the objective of which is to explore the convergence of nutritional elements, psychosocial factors, and demographic characteristics within a substantial IBD cohort and a control population exhibiting healthy characteristics. Subgroup and subanalysis findings from this study have been previously published [19–21]. The present study was designed to adhere to the ethical standards outlined in the Declaration of Helsinki (2013) concerning the conduct of research in human subjects. This monocentric study was conducted at a specialized medical facility and has been reviewed and approved by the Ethics Committee of Hannover Medical School (10847_BO_S_2023) and is duly documented in the German Clinical Trials Register (DRKS) under DRKS00032771.

### Participants and setting

From October 2023 to October 2024, a total of 275 patients with IBD were screened for enrollment at a tertiary referral center. Subjects meeting the study’s eligibility criteria, which included a confirmed diagnosis of ulcerative colitis or Crohn’s disease with a disease duration of at least three months, were included in the study. Informed consent, obtained through written means, was a prerequisite for inclusion in the study. Patients who were unwilling or unable to complete the screening visit, as well as individuals below the age of 18 or over the age of 90, were deemed ineligible for study participation. Patients with active malignant diseases were excluded from study participation. Pregnant patients as well as all patients with a therapeutic IBD diet were not screened for study participation.

**Fig. 1:**
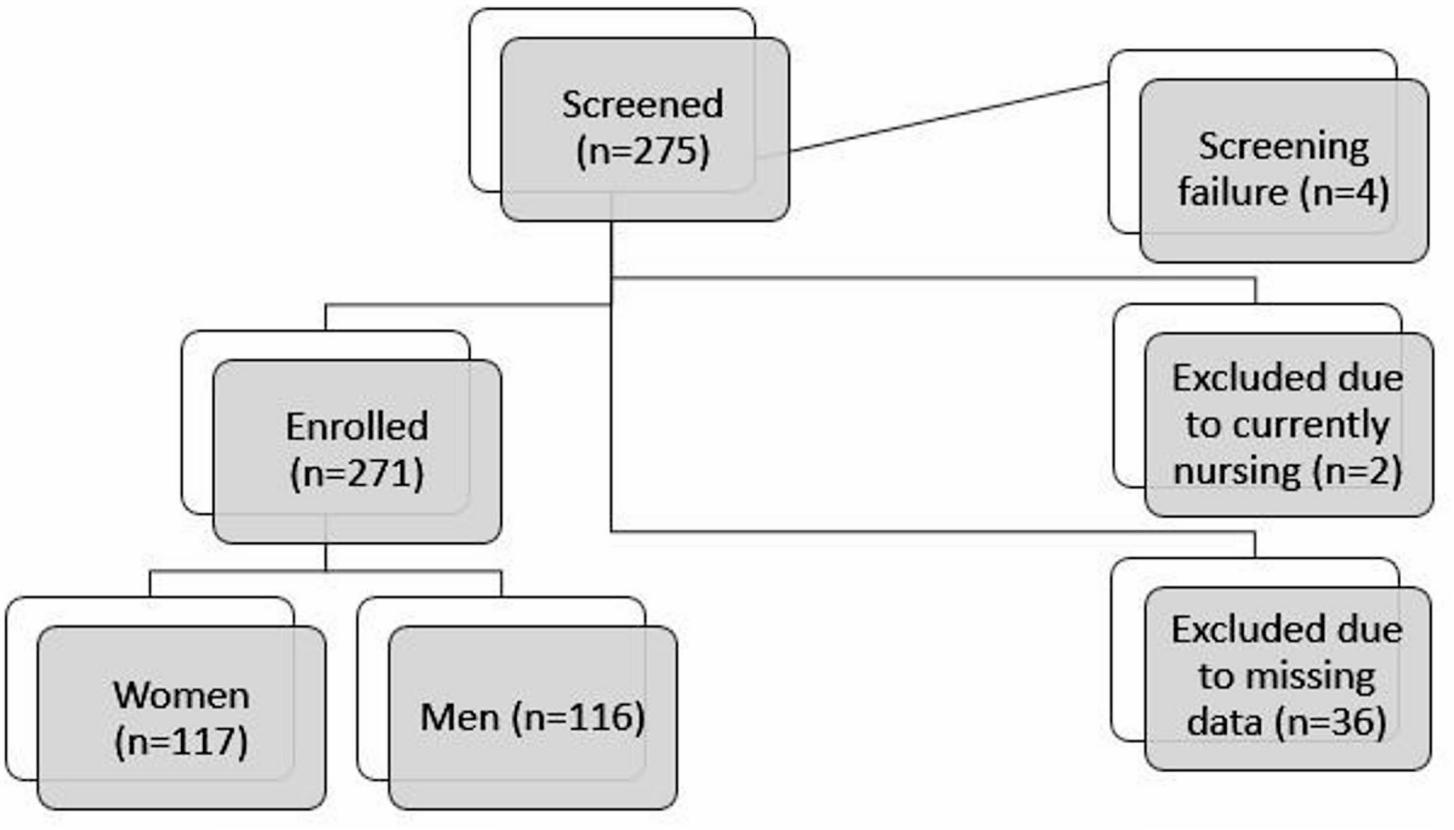
IBD cohort enrollment flow chart

In the cohort of 275 individuals diagnosed with IBD who underwent screening, four cases were identified as failing to meet the established enrollment criteria. Consequently, 271 individuals with IBD were enrolled in the study. Overall, 36 participants were omitted from the present analysis due to an absence of requisite dietary data. Moreover, two subjects were excluded from the study due to active nursing (Fig. 1).

#### Control cohort

The study’s control participants were drawn from a sample size of 101 local community-dwelling individuals, with written informed consent a prerequisite for inclusion. The participants of the control cohort were predominantly recruited from hospital staff and the local university student population. Furthermore, the study was publicised on the hospital homepage and volunteers were sought. Individuals diagnosed with IBD were excluded from enrollment in the control cohort. Individuals under the age of 18 or over the age of 90 did not meet the study’s eligibility requirements. Overall, three subjects were excluded from further analysis due to an absence of requisite dietary data, and two subjects were excluded due to nursing.

### Data sources/measurements

The study participants were instructed to complete an online survey, which included a series of questionnaires. This included a demographic survey, which captured data on subjects’ sex, age, body mass, and height, as well as information pertaining to comorbid psychiatric diagnoses, including, but not limited to, depression, anxiety, and burnout. In the context of logistic regression, a binary variable was developed to identify all who had been diagnosed with at least one of the specified diagnoses and were classified as probable cases of psychiatric distress. The survey’s accessibility was restricted exclusively to study participants who had previously provided written consent. In addition to the collection of demographic data, a validated food frequency questionnaire (FFQ), which was previously developed for the German Health Examination Survey for Adults (DEGS), was utilized [22]. The FFQ was administered to ascertain habitual dietary intake data over the last four weeks. The data was subsequently subjected to a mean-based calculation to derive the daily consumption amounts of particular foods and beverages [23]. The assessment of nutrient intake utilized Federal Food Code (BLS) reference data [24]. The estimated energy intake (EEI) and the sex-specific resting energy expenditure (REE) are expressed in kilojoules (kJ). The FFQ was further used to calculate the Mediterranean Diet Score (MDS), as adapted from Trichopoulou et al. [25]. The methodological approach employed to determine the MDS in the present study has been thoroughly delineated previously [26]. The proportion of energy derived from highly processed food was calculated using the German version of the Screening Questionnaire of Highly Processed Food Consumption—hereafter referred to as the sQ-HPF [27, 28]. All participants were additionally screened for malnutrition risk using the German version of the Malnutrition Universal Screening Tool (MUST) [29, 30]. IBD subjects were further asked to complete the German version of the FR-QoL-29, which is a questionnaire designed to evaluate food-related quality of life in IBD [19, 31]. Additionally, the web-based survey comprised inquiries regarding the patients’ individualized histories of IBD, the therapeutic regimens they have undergone, their surgical histories, and comorbidities. The IBD disease location and behavior was classified using two classification systems: first, the Montreal classification for Crohn’s disease, and second, the anatomical pattern classification for ulcerative colitis [32].

## Variables

### FACIT-fatigue

A PROMIS License for Individual Investigators to use the German version of the PROMIS^®^ Item Bank v1.0—Fatigue—Short Form 13a (FACIT-Fatigue) [18, 33, 34], was obtained from FACIT.ORG prior to study initiation. The FACIT-Fatigue scale is a psychometric instrument designed to evaluate the impact of fatigue on individuals’ daily activities and function. This scale utilizes a set of 13 self-report items that aim to assess various aspects of fatigue, providing a comprehensive evaluation of its effects. The FACIT-Fatigue scale has been validated for patients with IBD [35] as well as for the general German speaking population [18].The score range is from 0 to 52 with lower scores being indicative of fatigue. In a variety of populations, a number of cutoffs have been proposed with a general consensus emerging emerged that a score below 30 is indicative of severe, or moderate to severe, fatigue [36–38] Consequently, 30 was utilised as a cutoff for the present analysis to categorise study participants into two Fatigue groups: those equal or above (*low*) and those below (*high*) this value.

### Disease activity

Determination of disease activity and remission was achieved through application of disease activity index cut-offs specific to the entity in question; remission was defined as a Harvey-Bradshaw Index (HBI) [39] of less than five in patients with Crohn’s disease (CD) or a partial Mayo score (PMS) [40] of zero or one in patients with ulcerative colitis (UC).

### Diet quality

The quality of the diet was determined by employing the dietary guidelines established by the German Nutrition Society (DGE) [41]. The DGE guidelines delineate the recommended amounts of specific food items to be consumed, and the diet’s adherence to these guidelines was determined by assigning a point value to each recommendation. If a subject’s diet was found to be in accordance with the recommended amounts, the diet was assigned a value of 1 point. Conversely, if the observed diet did not align with the recommended amounts, it was assigned a value of 0 points. The resulting diet quality score ranges from 0 to 12, with 12 points signifying optimal adherence to the DGE guidelines and the best possible diet quality.

### Diet diversity

The German Nutrition Society (DGE) [41] guidelines were also employed to calculate the dietary diversity score. The scoring system is based on the daily or weekly consumption of the food items or food groups enumerated in the guidelines. These food items include fruits and vegetables, juice, beans and legumes, nuts and seeds, potatoes, butter and margarine, dairy products, fish, meat and poultry, deli meats, eggs, and cereals. Weekly data was converted to daily data and each instance of consumption of a given food or food group was assigned a point value, with the total points allocated to each participant determining their dietary diversity score. A diet characterized by significant variety was awarded a higher point value, e.g., 12 points for a highly diverse diet, whereas a diet lacking in variety received a lower point value, e.g., 1 point for a diet with minimal variety.

### Short health scale

The German version of the Short Health Scale (SHS) [42] was utilized to evaluate the current health status of individuals with IBD via a series of four inquiries concerning symptoms, daily activities, disease-related concerns, and general well-being over the preceding week. The assessment of all four domains enables the attainment of a maximum score of 400 points, with higher scores denoting diminished health-related quality of life for individuals with IBD.

### Anthropometric measurements

Data regarding height and weight were collected, after which the body mass index (BMI) was subsequently calculated. Handgrip strength was measured in all study participants by means of a hand-held dynamometer (Lafayette Instrument, Lafayette, IN, USA, Model 01165 A). In the context of chronic fatigue syndrome (CFS), handgrip strength is employed as an objective metric to assess the severity of the disease [43]. In the present subanalysis, handgrip strength was therefore utilized as a quantifiable metric to validate the fatigue score, given the anticipated correlation between decreased handgrip strength and elevated fatigue levels.

### Laboratory values

During the course of the screening visit, biomaterials (blood and stool samples) were collected in accordance with the established protocol during routine outpatient clinic visits. The laboratory values included C-reactive protein (CRP), measured in milligrams per liter, fecal calprotectin (mg/kg),, ferritin (µg/l), and hemoglobin (g/dl). For the control cohort, only Vitamin D3 25-OH serum levels were measured.

## Statistical analysis

Statistical analysis was conducted via the SPSS Statistics software, version 29.0.1.0 (SPSS, IBM, Armonk, NY), and GraphPad PRISM, version 10.4.0 (GraphPad Soft-ware, Boston, Massachusetts, USA). Categorical outcomes are reported as totals and proportions. The Shapiro-Wilks test was utilized to evaluate the normality of the data distribution. Continuous data is shown as mean (SD) and in instances where data is non-normally distributed, the median and interquartile range (IQR) are presented, with the exception of dietary data and patterns scores. In such instances, the application of the median would result in zero values for items with low consumption, thereby compromising the integrity of the findings. The statistical significance of the baseline characteristic variables was ascertained using either a student’s t-test, chi-square test, or Fisher’s exact test, with a Bonferroni correction employed where applicable. Unless otherwise specified, all statistical tests were two-sided. The significance levels are denoted as follows: one asterisk indicates *p* = 0.05, two asterisks indicate *p* = 0.01, and three asterisks indicate *p* < 0.001. In addition to statistical significance (*p*), the estimated effect size (ES) is reported as (g) for student’s t-test, (r) for Mann-Whitney-U test, and phi coefficient (rφ) for Fisher’s exact test. Student’s t-test and Mann Whitney u test were used according to variable distribution, for between group, and in-group comparisons. In cases involving substantial group size variation, the Mann-Whitney-U test was also implemented as the statistical method of choice. For the sex-stratified IBD cohort, a further analysis of fatigue and potential predictors was conducted via logistic regression. The results are reported as odds ratio (OR), the 95% confidence interval (CI) and the level of significance (*p*). The adjusted logistic regression model for women with IBD demonstrated a significantly superior fit in comparison to the intercept-only model (Omnibus test of model coefficients: *p* < 0.001). The goodness of fit was further evaluated using R2 (Nagelkerkes: 0.294; Cox&Snell: 0.212) and the Hosmer-Lemeshow test (*p* = 0.463). Model performance demonstrated an overall percentage of 79.1% on the classification table. Likewise, the adjusted logistic regression model for men with IBD demonstrated a significantly superior fit in comparison to the intercept-only model (Omnibus test of model coefficients: *p* < 0.001). The goodness of fit was further evaluated using R2 (Nagelkerkes: 0.334; Cox&Snell: 0.250) and the Hosmer-Lemeshow test (*p* = 0.784). Model performance demonstrated an overall percentage of 71.3% on the classification table. Adjustment factors for the fully adjusted models are sQ-HPF (%), water intake (l/d), age, BMI, psychiatric distress, protein intake, ferritin, hemoglobin, disease activity, diet diversity, diet quality, disease entity.

### Confounders and bias

Prior to the analysis of the data, cases were screened for individuals who were currently nursing. This screening revealed two individuals with IBD and two individuals of the control cohort who were actively nursing during their participation in the study. It was observed that the dietary intake of the nursing individuals was significantly higher. To avoid the potential distortion of the subsequent analysis, the data from all nursing individuals were consequently excluded. Furthermore, to exclude long-covid as a potential confounder for fatigue [44], all study participants were screened for long-covid. Altogether ten individuals (of which *n* = 1 CC) were diagnosed with long covid, while 257 study participants (78.1%) had a previous SARS-CoV-2 infection but no long-covid, and 62 study participants (18.8%) had no prior SARS-CoV-2 infection. All IBD cases with long covid were excluded from the logistic regression analysis. It is imperative to acknowledge the inherent limitations of recall surveys, which are susceptible to bias. Moreover, the underreporting of dietary intake in patient-reported outcomes is a pervasive issue [45]. In order to address this limitation, the extent of underreporting among study participants was determined by calculating the EEI-to-REE ratio, described before [26]. In addition, participants were questioned about whether they had initiated a new dietary regimen or modified their existing dietary habits within the five weeks preceding their inclusion in the study. The implementation of this inquiry aimed to identify any potential discrepancies between BMI and EEI. The control cohort exhibited an above-average percentage of individuals with higher education, and nearly 100% of those individuals were employed. This demographic is not representative of the broader German population, and it may introduce a confounding factor into the analysis since eating habits can differ between educational groups, with a shift towards more health-conscious eating among those with higher levels of education [46, 47]. In order to assess this possible limitation, a comparison was made between diet quality and diet diversity metrics from the control cohort and the IBD cohort. The control cohort demonstrated a lower diet diversity but a higher diet quality in comparison to the IBD cohort, independent of fatigue. Further analysis was therefore deemed possible.

In instances where single values were missing, it was assumed to be missing at random and omitted on an analysis-by-analysis basis.

## Results

### IBD cohort

The sex distribution demonstrated a balanced proportion, with 50.2% of the participants being women. Median age for women was 38 years, and 40 years for men. The body mass index was balanced between the sexes with a lower BMI reported for women (23.8) compared to men (24.4). Concurrently, the bulk of subjects in the study exhibited minimal probability of malnutrition (women: 48.7%; men: 61.2%), yet a comparatively higher percentage of patients were identified as being at high risk for malnutrition (women: 28.2%; men: 19.8%), as opposed to exhibiting medium risk (women: 23.1%; men: 19.0%). Estimated energy intake (kJ/d) exhibited expected sex-differences with women reporting an average of 6433 kJ per day compared to an average EEI of 7951 for men. A skewed distribution was further observed in terms of disease entities, with the majority of cases diagnosed as Crohn’s disease for both women (64.1%) and men (56.9%). The remission rate however demonstrated equilibrium, with 52.7% of women with IBD demonstrating remission, in comparison to 53.2% of men with IBD (Table 1).

**Table 1:** Demographic data of the IBD cohort

		Women	Men	
		(*n* = 117)	(*n* = 116)	*p*
Crohn’s disease [n(%)]		75 (64.1%)	66 (56.9%)	0.285
Current advanced drug therapy [n (%)]		65 (57%)	65 (57%)	0.999
Disease activity [n (%)]	Remission	58 (52.7%)	59 (53.2%)	0.999
Location of Crohn’s [n (%)]	L1: ileal	17 (22.7%)	18 (27.3%)	0.999
	L2: colonic	18 (24%)	7 (10.6%)	0.302
	L3: ileocolonic	32 (42.7%)	35 (53%)	0.999
	L4: isolated upper disease	8 (10.7%)	6 (9.1%)	0.999
Crohn’s behavior [n (%)]	B1: nonstricturing, nonpenetrating	31 (41.3%)	20 (30.3%)	0.999
	B2: stricturing	34 (45.3%)	32 (48.5%)	0.999
	B3: penetrating	10 (13.3%)	14 (21.2%)	0.999
UC Montreal classification [n (%)]	proctitis	3 (7.1%)	3 (6%)	0.999
	left-sided colitis	14 (33.3%)	18 (36%)	0.999
	pancolitis	25 (59.5%)	29 (58%)	0.999
Disease duration [median (IQR)] (years)		12 [7–20]	13 [7–19]	0.679
Surgery [n (%)]		39 (33.3%)	46 (39.7%)	0.343
Calprotectin [median (IQR)] (mg/kg)		82.3 [24.7–334]	129 [30.8–795]	0.438
C-reactive protein [median (IQR)] (mg/l)		2.1 [0.9–5.5]	1.4 [0.6–3.7]	0.514
Age [median (IQR)] (yrs)		38 [30–50]	40 [29–53]	0.843
MUST [n (%)]	low risk	57 (48.7%)	71 (61.2%)	0.332
	medium risk	27 (23.1%)	22 (19%)	0.999
	high risk	33 (28.2%)	23 (19.8%)	0.808
Education [n (%)]	Highschool diploma or higher	53 (45.3%)	64 (55.2%)	0.150
Work status [n (%)]	Currently employed/working	90 (76.9%)	98 (84.5%)	0.184
Vitamin D3 25-OH [median (IQR)] (ng/ml)		30 [21.8–37.1]	24.6 [20.8–34]	0.364
Handgripstrength [median (IQR)]		28.9 [23.3–33.4]	46.8 [38.6–54.4]	< 0.001
EEI [median (IQR)] (kJ/d)		6433 [4770–8952]	7951 [5797–11100]	0.004
BMI [median (IQR)] (kg/m^2^)		23.8 [21.5–28]	24.4 [21.2–27.8]	0.895

Data is reported as totals and proportions [n (%)] or median and interquartile range [Md (IQR)]. Statistical significance of the baseline characteristic variables was ascertained using either a student’s t-test, chi-square test, or Fisher’s exact test, with a Bonferroni correction employed where applicable. UC, ulcerative colitis; MUST, malnutrition universal screening tool; EEI, estimated energy intake; BMI, body mass index; kJ, kilojoule.

#### Control cohort

The distribution of participants according to sex was skewed (women *n* = 67 (69.8%)), the median age of women was 28 years [IQR: 23–45] and 32 for men [IQR: 24–37]. For women a lower BMI was observed (21.8) compared to men (24.8). As in the IBD cohort the EEI (kJ/d) showed expected sex-differences with women reporting an average of 6281 (kJ/d) compared to 8703 (kJ/d) for men. Most women of the control cohort exhibited a medium risk for malnutrition (*n* = 29 [43.3%]) whereas the majority of men exhibited a low risk of malnutrition (*n* = 18 [62.1%]) (Suppl. Table 1).

### Inflammatory bowel disease cohort versus control cohort

**Fig. 2:**
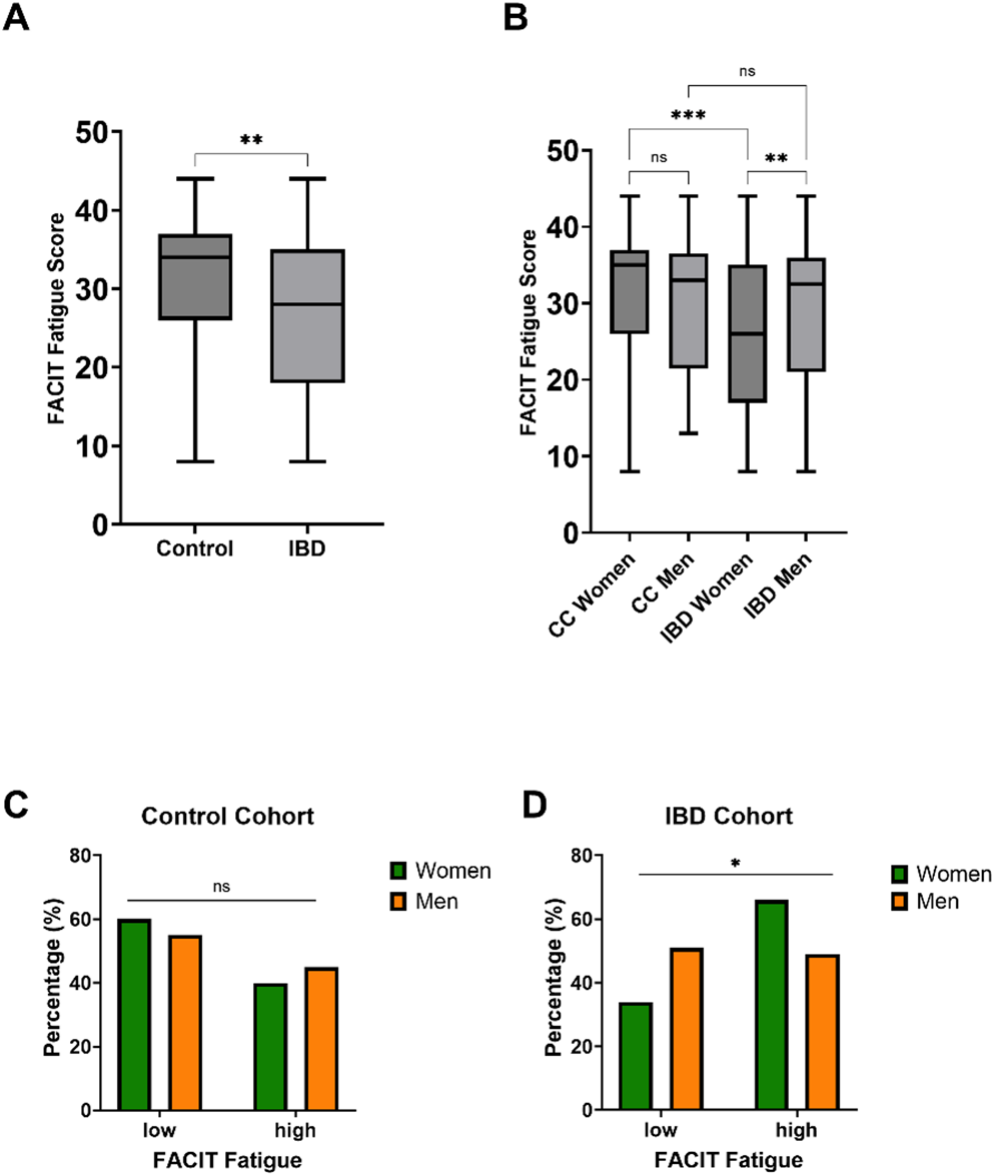
A-D fatigue comparison between the sexes and the cohorts. **A** the FACIT sum score shows statistically significant differences between the cohorts (*p* = 0.005; g = 0.3) with a clear trend towards a higher mean FACIT sum score for the control cohort; **B** no significant difference in mean FACIT sum score was observed between the sexes of the control cohort (women: 31; men: 30; *p* = 0.694; g = 1). However, for the IBD cohort the mean FACIT sum score was significantly lower for women compared to men (25 to 29; *p* = 0.009; g = − 0.3). FACIT sum score showed significant differences between women with IBD and women of the control cohort (*p* < 0.001; g = 0.5) but not for men (*p* = 0.684; g = 0.1); **C** binary distribution of FACIT sum score was not statistically different between men and women in the control cohort (*p* = 0.822; rφ = 0); **D** between men and women with IBD binary distribution of FACIT sum was statistically significant (*p* = 0.012; rφ = − 0.2). The significance levels are denoted as follows: one asterisk indicates *p* = 0.05, two asterisks indicate *p* = 0.01, and three asterisks indicate *p* < 0.001. IBD—inflammatory bowel disease; CC—control cohort; ns—not significant; low—FACIT-fatigue sum score of 30 or more, indicating none to mild fatigue; high—FACIT-fatigue sum score of 29 or less, indicating moderate to severe fatigue.

In order to examine cohort-specific differences in fatigue, as measured by the FACIT sum score, student t-test was used. The FACIT sum score shows statistically significant differences between the cohorts (*p* = 0.005; g = 0.3) with a clear trend towards a higher mean FACIT sum score for the control cohort (Fig. 2a). In order to examine sex-differences in the FACIT sum score student’s t-test was used. For the control cohort no significant difference in mean FACIT sum score was observed between the sexes (women: 31; men: 30; *p* = 0.694; g = 1). However, for the IBD cohort the mean FACIT sum score was significantly lower for women compared to men (25 to 29; *p* = 0.009; g = − 0.3) (Fig. 2b). Binary distribution of FACIT sum score was not statistically different between men and women in the control cohort (*p* = 0.822; rφ = 0) (Fig. 2c). However, between men and women with IBD the distribution was statistically significant (*p* = 0.012; rφ = − 0.2) (Fig. 2d).

### Fatigue and IBD

This section focuses exclusively on the analysis of the IBD cohort, stratified by sex.

#### Fatigue levels and dietary pattern scores

Possible sex-related differences as well as IBD-specific differences in dietary patterns and fatigue were investigated via student’s t-test. No statistically significant differences were detected in the fatigue groups among women; however, a statistically significant difference was observed in the Mediterranean diet score (MDS) between the fatigue groups among men (*p* = 0.016; g = 0.5). The MDS was significantly different between men and women in the low fatigue group (*p* = 0.041; g = − 0.4). In both the low and the high fatigue group, significant differences in diet quality between the sexes were observed (low: *p* = 0.006; g = 0.6; high: *p* = 0.014; g = 0.4) (Table 2). In addition, a granular analysis of possible sex-related differences as well as cohort-specific differences in dietary patterns, dietary choices, and macronutrients in comparison with fatigue showed notable distinctions between the sexes and within the distinct cohorts examined in the study. For one, the comparative cohort analysis demonstrated a prevailing trend of suboptimal dietary habits among individuals diagnosed with IBD when juxtaposed with the control group (Suppl. Table 2).

**Table 2:** Fatigue levels and IBD

				Women							Men					Sex by fatigue	
		Fatigue	n	mean	SD	SEM	*p*	g		n	mean	SD	SEM	*p*	g	*p*	g
SHS		low	37	142.9	120.2	19.8	**< 0.001**	− 0.8		56	119.7	91.4	12.2	**< 0.001**	− 0.7	0.295	0.2
		high	75	230.6	105	12.1				57	187.9	109.1	14.4			**0.025**	0.4
FR-QoL-29		low	40	89.3	24	3.8	**0.004**	0.6		59	91.7	20.8	2.7	**0.018**	0.4	0.600	− 0.1
		high	77	77	20.2	2.3				57	82.6	20	2.7			0.113	− 0.3
Diet Quality		low	40	5.4	1.4	0.2	0.592	− 0.1		59	4.5	1.6	0.2	0.154	− 0.3	**0.006**	0.6
		high	77	5.6	1.6	0.2				57	4.9	1.4	0.2			**0.014**	0.4
Diet Diversity		low	40	5.5	1.3	0.2	0.986	0		59	5.9	1.4	0.2	0.546	0.1	0.089	− 0.3
		high	77	5.5	1.4	0.2				57	5.8	1.4	0.2			0.192	− 0.2
MDS		low	40	4	1.6	0.2	0.446	0.1		59	4.7	1.5	0.2	**0.016**	0.5	**0.041**	− 0.4
		high	77	3.8	1.7	0.2				57	4	1.6	0.2			0.522	− 0.1
sQ-HPF (%)		low	40	36.6	5.7	0.9	0.511	0.1		59	36.9	6.3	0.8	0.658	− 0.1	0.786	− 0.1
		high	77	35.7	7.6	0.9				57	37.4	6.6	0.9			0.162	− 0.2
Calprotectin (mg/kg)		low	34	248.6	618.7	106.1	**0.006**	− 0.4		50	692.4	1592	225.2	0.190	− 0.3	0.079	− 0.3
		high	71	991.1	2048	243				53	1191	2177	299.1			0.602	− 0.1
C-reactive protein (mg/l)		low	36	3.5	4.2	0.7	**0.034**	− 0.3		52	4.9	10.9	1.5	0.711	− 0.1	0.476	− 0.2
		high	75	7.9	16.3	1.9				53	5.7	12	1.6			0.422	0.1

Results of student’s t-test comparison for sex-differences in dietary patterns, IBD-related quality of life, and inflammation parameters in comparison with fatigue. Significant findings (p≤0.05) are denoted in bold. SHS, short health scale; FR-QoL-29, food-related quality of life questionnaire; MDS, mediterranean diet score; sQ-HPF, screening questionnaire of highly processed food consumption; SD, standard deviation; SEM, standard error of the mean.

#### Fatigue levels and disease parameters

Sex-related differences for objective disease parameters and fatigue were assessed via student’s t-test. Significant differences between women with low and high fatigue for fecal calprotectin (*p* = 0.006; g = − 0.4) and c-reactive protein (*p* = 0.034; g = − 0.3) were observed. No statistically significant differences were detected for men or between the sexes (Table 2).

#### Fatigue levels and psychosocial proms

Possible sex-related differences as well as IBD-specific differences in psychosocial PROMs and fatigue were further investigated via student’s t-test. A statistically significant discrepancy was identified in the health-related quality of life, as measured by the SHS, between the fatigue levels for both men (*p* < 0.001; g = − 0.7) and women (*p* < 0.001; g = − 0.8). A subsequent analysis of the data revealed that this disparity remained statistically significant only for the high fatigue group (*p* = 0.025; g = 0.4). Generally, the health-related quality of life of women with low and high fatigue was found to be lower than that of men. Additionally, a statistically significant difference was observed in IBD food-related quality of life based on fatigue levels for both men (*p* = 0.018; g = 0.4) and women (*p* = 0.004; g = 0.6), but no significant difference was observed between the sexes. However, women with low and high fatigue also exhibited a consistently lower IBD food-related quality of life compared to men (Table 2).

#### Logistic regression of factors associated with fatigue in men and women with IBD

To identify possible risk factors for high fatigue adjusted multivariable logistic regression analysis was conducted for men with IBD and women with IBD respectively which demonstrated distinct influences within the sexes. For women active disease was identified as a risk factor [OR: 7.8 (95% CI 2.44–23.56)] whereas age appears to mitigate the risk of fatigue [OR: 0.9 (95% CI 0.91–0.98)] (Table 3). Meanwhile, for men active disease [OR: 3.5 (95% CI 1.19–10.40)] as well as psychiatric distress [OR: 4.1 (95% CI 1.06–16.36)] were identified as risk factors, while daily water intake [OR: 0.7 (95% CI 0.53–0.96)] and a diverse diet [OR: 0.6 (95% CI 0.45–0.96)] appear to mitigate the risk of fatigue (Table 4).

**Table 3:** Logistic regression of factors associated with fatigue in women with IBD

Predictor		Category/unit		OR		95% CI for OR		*p*
Intercept		–		6.965		–		0.010
Age		Years		0.952		0.919–0.986		0.006
Disease activity		Active disease		7.859		2.445–23.561		< 0.001

Logistic regression analysis results are reported as odds ratio (OR), the 95% confidence interval (CI) and the level of significance (p). Adjustment factors for the fully adjusted models are sQ-HPF (%), water intake (l/d), age, BMI, psychiatric distress, protein intake, ferritin, hemoglobin, disease activity, diet diversity, diet quality, disease entity.

**Table 4:** Logistic regression of factors associated with fatigue in men with IBD

Predictor	Category/unit	OR	95% CI for OR	*p*
Intercept	–	1.135	–	0.937
Water intake	d/l	0.718	0.533–0.967	0.029
BMI	kg/m^2^	1.088	0.983–1.205	0.103
Psychiatric distress	yes	4.184	1.069–16.369	0.040
Disease activity	Active disease	3.524	1.194–10.400	0.023
Diet diversity	Score	0.664	0.457–0.964	0.031

Logistic regression analysis results are reported as odds ratio (OR), the 95% confidence interval (CI) and the level of significance (p). Adjustment factors for the fully adjusted models are sQ-HPF (%), water intake (l/d), age, BMI, psychiatric distress, protein intake, ferritin, hemoglobin, disease activity, diet diversity, diet quality, disease entity.

## Discussion

A paucity of research data exists concerning sex-differences in the context of IBD, particularly with regard to diet and distinct psychosocial aspects as fatigue. This study presents a thorough investigation of the dietary patterns and preferences exhibited by individuals diagnosed with IBD, with varying degrees of fatigue. To circumvent an underestimation of the potential influence of the disease burden, a comparative analysis was conducted with a control cohort. Analyses of anthropometric measures showed an expected shift in handgrip strengths between low and high fatigue groups for men and women with IBD as well as men and women of the control cohort thus additionally validating the fatigue score for these cohorts.

The comparative cohort analysis indicated a general trend of suboptimal dietary habits among individuals with IBD in comparison to the control cohort. This observation manifested as an elevated sQ-HPF percentage and a diminished diet quality. A particularly salient finding is the conspicuously elevated percentage of highly-processed food intake observed in all individuals with IBD, irrespective of their sex or level of fatigue. Contrasted with the control cohort, this finding was significant and is a cause of concern. This is in contrast to the established nutritional recommendations for patients with IBD, which advocate for a Mediterranean diet that is predominantly plant-based and minimal in ultra-processed foods [48, 49]. A more thorough analysis of the IBD cohort was undertaken. While the sex distribution in our IBD cohort was well balanced, sex-specific shifts in the fatigue groups were observed, as well as sex differences between the fatigue level groups. A comparative analysis of IBD-related quality of life as measured by the SHS, revealed that individuals with high fatigue exhibited significantly lower IBD related QoL compared to their counterparts with low fatigue. This was particularly true in women. Moreover, the presence of high fatigue in women with IBD led to a pronounced decline in their QoL, which was significantly lower than that observed in men with IBD. This is consistent with findings by Jonefjäll et al., arguing the direction of influence goes from fatigue to QoL [50] and suggests a more pronounced disease burden for women. Furthermore, a substantial discrepancy was observed in the IBD-specific food-related quality of life among the fatigue groups of men and women. In this case as well, women across all groups exhibited a diminished quality of life, accompanied by elevated levels of fatigue. Therefore, the overall findings of our analysis indicate that the disease burden of IBD appears to manifest with more significant psychosocial implications for women than for men. This observation is consistent with the findings reported in recent research [51]. Further explanations for this sex-specific finding could be hypothesised as, research findings from diverse populations indicate that social context exerts a substantial influence on the experience of fatigue among women, affecting both the manifestation and management of this condition with a notable aspect of this influence pertains to caregiving expectations, which, as indicated by research, are disproportionately placed upon women. Additionally, the interplay between professional and domestic responsibilities has been demonstrated to influence women’s fatigue levels for the worse [17, 52, 53]. Those aspects have the potential to intensify the experience of fatigue. Taking into account the further results of our analysis, that showed that in addition to the association of poor IBD-related quality of life with severe fatigue, objective measures of disease (fecal calprotectin) and inflammation (CRP) exhibited a substantial sex-specific shift between the fatigue groups in women but not in men, fatigue in women with IBD seems to be influenced by an interplay of social and disease burden. This was underlined by the analysis of women in the control cohort which revealed no substantial variance in fatigue ratings when compared to men of the same cohort. Additionally, a higher proportion of women in the control group fall into the category of low fatigue compared to the category of high fatigue, contrasting with the observed pattern in the IBD group. Overall, this suggests that women with IBD are significantly more affected by fatigue than their counterparts in the control cohort, indicating that the disease burden on women diagnosed with IBD is notably heavier. Meanwhile, we suggest that men with IBD seem to exhibit a greater degree of adaptability in their dietary behavior compared to women with IBD as the present study identified that a diet comprising a variety of foods and adequate daily water intake appeared to mitigate the risk of high fatigue in men with IBD. This is in line with previous findings which demonstrated that men with IBD exhibited a shift in dietary patterns in association with disease-related QoL [26]. 

### Limitations

 The present study acknowledges its own limitations. For one, no body composition data is available. Moreover, in order to execute a more comprehensive dietary behaviour analysis, it is necessary to employ 24 h recall surveys and dietary diaries. This is because such methods allow for the adjustment of underreporting and the consideration of specialised diets, such as vegan diets, which are frequently not accounted for in FFQs. Moreover, while the analysis was adjusted for possible fatigue risk such as disease activity, psychiatric distress, low water and protein intake, and anemia, data on further fatigue risk factors was not available. This includes duration of remission, sleep disturbance, pain and physical activity and sedentary behavior. The interplay between fatigue and physical activity exhibits a bidirectional dynamic and cannot be further answered in this study. While it is well-documented that engaging in physical activity can serve as a counteragent to fatigue, it is equally important to acknowledge the reciprocal effect of fatigue on physical activity participation [54].

Overall, the present analysis was conducted as part of a cross-sectional study. It is therefore evident that further studies are necessary in order to conduct a more targeted analysis and to address the limitations of the present analysis.

## Supplementary Information

Below is the link to the electronic supplementary material.

Supplementary Material 1.

Supplementary Material 2.

## Data Availability

The original contributions presented in the study are included in the article, further inquiries can be directed to the corresponding author.
