# Supplementary material for: Disparities in fatigue levels and dietary habits between men and women with inflammatory bowel disease: a comparative analysis with a control cohort
Source: Eur J Nutr. 2026 Feb 16;65(2):59. doi: 10.1007/s00394-026-03902-2 (PMC12909621; doi:10.1007/s00394-026-03902-2)
Supplement: Supplementary file 1 — Supplementary Material 1. [file 394_2026_3902_MOESM1_ESM.docx]

Supplementary Table 1 Demographic data of the control cohort.

|  |  | Women | Men |  |
| --- | --- | --- | --- | --- |
|  |  | (n=67) | (n=29) | *p* |
| MUST [n (%)] | low risk | 24 (35.8%) | 18 (62.1%) | 0.104 |
|  |  |  |  |  |
|  | medium risk | 29 (43.3%) | 4 (13.8%) | **0.031** |
|  |  |  |  |  |
|  | high risk | 14 (20.9%) | 7 (24.1%) | 0.999 |
|  |  |  |  |  |
| Education [n (%)] | Highschool Diploma or higher | 49 (73.1%) | 23 (79.3%) | 0.614 |
|  | |  |  |  |
| Work status [n (%)] | Currently employed/working | 66 (98.5%) | 29 (100%) | 0.999 |
|  |  |  |  |  |
| Vitamin D3 25-OH [median (IQR)] (ng/ml) | | 26.3 [20.8 - 33.4] | 18.3 [14.9 - 26.6] | **0.004** |
|  |  |  |  |  |
| Age [median (IQR)] (yrs) |  | 28 [23 - 45] | 32 [24 - 37] | 0.764 |
|  |  |  |  |  |
| Handgripstrength [median (IQR)] | | 33.1 [29.3 - 38.6] | 52.6 [45.4 - 62.7] | **<0.001** |
|  |  |  |  |  |
| EEI [median (IQR)] (kJ/d) |  | 6281 [4688 - 7604] | 8703 [6785 - 9798] | **<0.001** |
|  |  |  |  |  |
| BMI [median (IQR)] (kg/m^2^) |  | 21.8 [20.3 - 25.5] | 24.8 [22.6 - 26.5] | 0.177 |
| Data is reported as totals and proportions [n (%)] or median and interquartile range [Md (IQR)]. Statistical significance of the baseline characteristic variables was ascertained using either a student’s t-test, chi-square test, or Fisher's exact test, with a Bonferroni correction employed where applicable. MUST – malnutrition universal screening tool; EEI – estimated energy intake; BMI – body mass index; kJ – kilojoule. | | | | |
