# Supplementary material for: Disparities in fatigue levels and dietary habits between men and women with inflammatory bowel disease: a comparative analysis with a control cohort
Source: Eur J Nutr. 2026 Feb 16;65(2):59. doi: 10.1007/s00394-026-03902-2 (PMC12909621; doi:10.1007/s00394-026-03902-2)
Supplement: Supplementary file 2 — Supplementary Material 2. [file 394_2026_3902_MOESM2_ESM.docx]

Supplementary Table 2 Fatigue levels and diet in IBD men and women and a control cohort.

|  |  |  |  |  |  |  |  |  |  |  |  |  |  |  |  |  |  |  |  |  |  |  |  |  |  |  |  |  |  |  |  |  |  |  |  |  |
| --- | --- | --- | --- | --- | --- | --- | --- | --- | --- | --- | --- | --- | --- | --- | --- | --- | --- | --- | --- | --- | --- | --- | --- | --- | --- | --- | --- | --- | --- | --- | --- | --- | --- | --- | --- | --- |
|  |  |  | **Fatigue (low = >/= 30)** | | | | | | | | | | | | | | | |  | **Fatigue (high = <30)** | | | | | | | | | | | | | | | |  |
|  |  |  |  |  |  |  |  |  |  |  |  |  |  |  |  |  |  |  |  |  |  |  |  |  |  |  |  |  |  |  |  |  |  |  |  |  |
|  |  |  |  | **IBD** | | | |  |  |  | **CC** | | | |  |  | **IBD vs CC** | |  |  | **IBD** | | | |  |  |  | **CC** | | | |  |  | **IBD vs CC** | |  |
|  |  | Sex | n | mean | SD | SEM | p | effect size |  | n | mean | SD | SEM | p | effect size |  | p | effect size |  | n | mean | SD | SEM | p | effect size |  | n | mean | SD | SEM | p | effect size |  | p | effect size |  |
| Handgrip strength | | women | 38 | 30.3 | 5 | 0.8 | **<0.001** | -1.9 |  | 40 | 34.5 | 6.7 | 1.1 | **<0.001** | -0.6* |  | **0.003** | 0.7 |  | 77 | 28.4 | 8.5 | 1 | **<0.001** | -1.7 |  | 26 | 32.4 | 8.2 | 1.6 | **<0.001** | -2.4 |  | **0.020** | -0.2* |  |
|  |  | men | 59 | 48.8 | 11.4 | 1.5 |  |  |  | 16 | 53.4 | 14.4 | 3.6 |  |  |  | 0.170 | -0.2* |  | 55 | 46.3 | 13 | 1.7 |  |  |  | 12 | 53.2 | 9.2 | 2.7 |  |  |  | **0.035** | -0.3* |  |
| Body weight (kg) | | women | 40 | 69.1 | 12.4 | 2 | **<0.001** | -0.8 |  | 40 | 64.7 | 12.8 | 2 | **<0.001** | -1.5 |  | 0.125 | -0.3 |  | 77 | 69.4 | 16.9 | 1.9 | **<0.001** | -0.8 |  | 27 | 68.6 | 16.4 | 3.2 | **0.004** | -1.0 |  | 0.770 | 0* |  |
|  |  | men | 59 | 82.8 | 19.6 | 2.6 |  |  |  | 16 | 85.3 | 15.3 | 3.8 |  |  |  | 0.375 | -0.1* |  | 57 | 83.6 | 19.3 | 2.6 |  |  |  | 13 | 83.4 | 8.9 | 2.5 |  |  |  | 0.994 | 0* |  |
| Vitamin D3 25-OH (ng/ml) | | women | 18 | 29.8 | 9.9 | 2.3 | 0.924 | 0 |  | 40 | 27.8 | 10.3 | 1.6 | **0.007** | 0.8 |  | 0.486 | -0.2 |  | 40 | 30.5 | 13.6 | 2.2 | 0.220 | 0.3 |  | 26 | 27.7 | 13.2 | 2.6 | 0.177 | 0.5 |  | 0.210 | -0.2* |  |
|  |  | men | 31 | 29.4 | 14.3 | 2.6 |  |  |  | 16 | 19.8 | 7.1 | 1.8 |  |  |  | **0.006** | -0.4* |  | 27 | 26.7 | 10 | 1.9 |  |  |  | 12 | 21.8 | 9.3 | 2.7 |  |  |  | 0.210 | -0.2* |  |
| Estimated energy intake (kJ/d) | | women | 40 | 7677 | 3958 | 625.8 | 0.131 | -0.3 |  | 40 | 6502 | 2469 | 390.4 | **<0.001** | -1.1 |  | 0.116 | -0.4 |  | 77 | 7150 | 3613 | 411.7 | **0.023** | -0.4 |  | 27 | 7126 | 2867 | 551.8 | 0.210 | -0.4 |  | 0.747 | 0* |  |
|  |  | men | 59 | 8929 | 4058 | 528.3 |  |  |  | 16 | 9395 | 3179 | 794.6 |  |  |  | 0.423 | -0.1* |  | 57 | 8810 | 4739 | 627.7 |  |  |  | 13 | 8286 | 2274 | 630.7 |  |  |  | 0.734 | 0* |  |
| Diet Quality (DGE) | | women | 40 | 5.4 | 1.4 | 0.2 | **0.006** | 0.6 |  | 40 | 6.5 | 1.8 | 0.3 | 0.202 | 0.4 |  | **0.004** | 0.7 |  | 77 | 5.6 | 1.6 | 0.2 | **0.014** | 0.4 |  | 27 | 6.8 | 1.8 | 0.3 | 0.393 | 0.3 |  | **0.003** | -0.3* |  |
|  |  | men | 59 | 4.5 | 1.6 | 0.2 |  |  |  | 16 | 5.8 | 1.6 | 0.4 |  |  |  | **0.014** | -0.3* |  | 57 | 4.9 | 1.4 | 0.2 |  |  |  | 13 | 6.3 | 1.7 | 0.5 |  |  |  | **0.007** | -0.3* |  |
| Diet Diversity (DGE) | | women | 40 | 5.5 | 1.3 | 0.2 | 0.089 | -0.3 |  | 40 | 4.9 | 1.6 | 0.3 | 0.889 | 0 |  | 0.082 | -0.4 |  | 77 | 5.5 | 1.4 | 0.2 | 0.192 | -0.2 |  | 27 | 5.1 | 1.5 | 0.3 | 0.944 | 0 |  | 0.166 | -0.1* |  |
|  |  | men | 59 | 5.9 | 1.4 | 0.2 |  |  |  | 16 | 4.8 | 1.3 | 0.3 |  |  |  | **0.004** | -0.3* |  | 57 | 5.8 | 1.4 | 0.2 |  |  |  | 13 | 5.1 | 1.4 | 0.4 |  |  |  | 0.112 | -0.2* |  |
| Mediterranean Diet Score | | women | 40 | 4 | 1.6 | 0.2 | **0.041** | -0.4 |  | 40 | 4.6 | 1.5 | 0.2 | 0.725 | -0.1 |  | 0.087 | 0.4 |  | 77 | 3.8 | 1.7 | 0.2 | 0.522 | -0.1 |  | 27 | 4.7 | 1.7 | 0.3 | 0.404 | 0.3 |  | **0.021** | -0.2* |  |
|  |  | men | 59 | 4.7 | 1.5 | 0.2 |  |  |  | 16 | 4.8 | 2.3 | 0.6 |  |  |  | 0.682 | 0* |  | 57 | 4 | 1.6 | 0.2 |  |  |  | 13 | 4.2 | 2.3 | 0.6 |  |  |  | 0.712 | 0* |  |
| sQ-HPF (%) | | women | 40 | 36.6 | 5.7 | 0.9 | 0.786 | -0.1 |  | 40 | 24.5 | 6.8 | 1.1 | **0.006** | -0.8 |  | **<0.001** | -1.9 |  | 77 | 35.7 | 7.6 | 0.9 | 0.162 | -0.2 |  | 27 | 25.8 | 6.8 | 1.3 | 0.287 | -0.4 |  | **<0.001** | -0.5* |  |
|  |  | men | 59 | 36.9 | 6.3 | 0.8 |  |  |  | 16 | 30.6 | 8.1 | 2 |  |  |  | **0.008** | -0.3* |  | 57 | 37.4 | 6.6 | 0.9 |  |  |  | 13 | 28.5 | 8.6 | 2.4 |  |  |  | **<0.001** | -0.4* |  |
| Carbohydrates (EN%) | | women | 40 | 50.7 | 8.3 | 1.3 | 0.793 | -0.1 |  | 40 | 50 | 7.6 | 1.2 | 0.129 | -0.4 |  | 0.684 | -0.1 |  | 77 | 51.7 | 9.9 | 1.1 | 0.394 | 0.1 |  | 27 | 51.6 | 6.6 | 1.3 | **0.010** | 0.9 |  | 0.973 | 0* |  |
|  |  | men | 59 | 51.2 | 9.2 | 1.2 |  |  |  | 16 | 53.1 | 4.6 | 1.1 |  |  |  | 0.137 | -0.2* |  | 57 | 50.4 | 8 | 1.1 |  |  |  | 13 | 44.7 | 9.1 | 2.5 |  |  |  | **0.024** | -0.3* |  |
| Fat (EN%) | | women | 40 | 31.2 | 7 | 1.1 | 0.921 | 0 |  | 40 | 30.6 | 5.9 | 0.9 | **0.030** | 0.6 |  | 0.674 | -0.1 |  | 77 | 31.2 | 7.8 | 0.9 | 0.876 | 0 |  | 27 | 29.9 | 5.6 | 1.1 | 0.068 | -0.6 |  | 0.322 | -0.1* |  |
|  |  | men | 59 | 31.4 | 7.2 | 0.9 |  |  |  | 16 | 27 | 4.3 | 1.1 |  |  |  | **0.008** | -0.3* |  | 57 | 31.4 | 6.6 | 0.9 |  |  |  | 13 | 33.3 | 4.6 | 1.3 |  |  |  | 0.254 | -0.1* |  |
| Protein (EN%) | | women | 40 | 16 | 3.9 | 0.6 | 0.398 | 0.2 |  | 40 | 17.1 | 4.1 | 0.7 | 0.873 | 0 |  | 0.212 | 0.3 |  | 77 | 15.4 | 4.1 | 0.5 | 0.412 | -0.1 |  | 27 | 16.4 | 3.4 | 0.7 | 0.233 | -0.4 |  | 0.063 | -0.2* |  |
|  |  | men | 59 | 15.4 | 3.1 | 0.4 |  |  |  | 16 | 17.3 | 2.9 | 0.7 |  |  |  | **0.037** | -0.2* |  | 57 | 15.9 | 3.3 | 0.4 |  |  |  | 13 | 17.9 | 4 | 1.1 |  |  |  | 0.197 | -0.2* |  |
| Animal protein (EN%) | | women | 40 | 9.4 | 4.2 | 0.7 | 0.712 | 0.1 |  | 40 | 9.4 | 4.9 | 0.8 | 0.885 | 0 |  | 0.968 | 0 |  | 77 | 8.9 | 4.7 | 0.5 | 0.145 | -0.3 |  | 27 | 7.9 | 3.2 | 0.6 | 0.064 | -0.7* |  | 0.581 | -0.1* |  |
|  |  | men | 59 | 9.1 | 3.4 | 0.4 |  |  |  | 16 | 9.6 | 3.7 | 0.9 |  |  |  | 0.866 | 0* |  | 57 | 10 | 3.7 | 0.5 |  |  |  | 13 | 10.7 | 5.5 | 1.5 |  |  |  | 0.723 | 0* |  |
| Sugar (EN%) | | women | 40 | 25.2 | 8.7 | 1.4 | 0.078 | 0.4 |  | 40 | 22.9 | 6.9 | 1.1 | 0.756 | 0.1 |  | 0.193 | -0.3 |  | 77 | 24.6 | 9.5 | 1.1 | 0.106 | 0.3 |  | 27 | 24.8 | 11.4 | 2.2 | **0.044** | 0.7 |  | 0.991 | 0* |  |
|  |  | men | 59 | 22 | 9 | 1.2 |  |  |  | 16 | 22.3 | 7.1 | 1.8 |  |  |  | 0.372 | -0.1* |  | 57 | 22 | 9 | 1.2 |  |  |  | 13 | 17.6 | 7 | 1.9 |  |  |  | 0.167 | -0.2* |  |
| Fruits and vegetables (EN%) | | women | 40 | 10.7 | 8.8 | 1.4 | **0.028** | 0.5 |  | 40 | 12.4 | 6.8 | 1.1 | 0.113 | 0.5 |  | 0.337 | 0.2 |  | 77 | 8.7 | 7.4 | 0.8 | **0.013** | 0.4 |  | 27 | 9.9 | 5.5 | 1.1 | 0.455 | 0.2 |  | 0.090 | -0.2* |  |
|  |  | men | 59 | 7.2 | 5.6 | 0.7 |  |  |  | 16 | 9.4 | 5 | 1.2 |  |  |  | **0.049** | -0.2* |  | 57 | 6 | 5.1 | 0.7 |  |  |  | 13 | 8.2 | 8.7 | 2.4 |  |  |  | 0.492 | -0.1* |  |
| Legumes and Pulses (EN%) | | women | 40 | 1.3 | 2.9 | 0.5 | 0.360 | 0.2 |  | 40 | 1.8 | 1.9 | 0.3 | 0.116 | -0.5 |  | 0.360 | 0.2 |  | 76 | 0.7 | 0.9 | 0.1 | 0.384 | 0.1 |  | 27 | 1.9 | 2.3 | 0.4 | 0.455 | -0.2 |  | **<0.001** | -0.3* |  |
|  |  | men | 58 | 0.9 | 1.1 | 0.1 |  |  |  | 16 | 2.8 | 2.4 | 0.6 |  |  |  | **<0.001** | -0.4* |  | 57 | 0.6 | 0.6 | 0.1 |  |  |  | 13 | 2.5 | 2.6 | 0.7 |  |  |  | **<0.001** | -0.4* |  |
| Nuts and seeds (EN%) | | women | 40 | 3.1 | 5.1 | 0.8 | 0.084 | 0.4 |  | 39 | 4.9 | 7.2 | 1.1 | 0.557 | 0.2 |  | 0.196 | 0.3 |  | 76 | 2.3 | 3.9 | 0.4 | 0.064 | 0.3 |  | 27 | 6.3 | 9.6 | 1.8 | 0.991 | 0 |  | **0.013** | -0.2* |  |
|  |  | men | 59 | 1.5 | 2.9 | 0.4 |  |  |  | 15 | 3.7 | 4.8 | 1.2 |  |  |  | 0.151 | -0.2* |  | 57 | 1.3 | 1.9 | 0.3 |  |  |  | 13 | 6.3 | 7.1 | 2 |  |  |  | **0.015** | -0.3* |  |
| Cereal products (EN%) | | women | 40 | 16.8 | 7.9 | 1.2 | **0.015** | -0.5 |  | 40 | 20 | 12.9 | 2 | 0.213 | -0.4 |  | 0.186 | 0.3 |  | 77 | 18.3 | 7.9 | 0.9 | 0.485 | -0.1 |  | 27 | 19 | 8.9 | 1.7 | 0.678 | 0.1 |  | 0.827 | 0* |  |
|  |  | men | 59 | 21.6 | 11.4 | 1.5 |  |  |  | 16 | 24.8 | 13.1 | 3.3 |  |  |  | 0.345 | -0.1* |  | 57 | 19.4 | 9.7 | 1.3 |  |  |  | 13 | 17.9 | 5.7 | 1.6 |  |  |  | 0.803 | 0* |  |
| Dairy products (EN%) | | women | 40 | 14.1 | 9.2 | 1.5 | 0.100 | 0.3 |  | 40 | 17.5 | 11 | 1.7 | 0.676 | 0.1 |  | 0.136 | 0.3 |  | 77 | 12.2 | 9 | 1 | 0.970 | 0 |  | 27 | 13.7 | 7.6 | 1.5 | 0.724 | 0.1 |  | 0.198 | -0.1* |  |
|  |  | men | 59 | 11.1 | 8 | 1 |  |  |  | 16 | 16.2 | 8.4 | 2.1 |  |  |  | **0.030** | -0.3* |  | 57 | 12.2 | 7.5 | 1 |  |  |  | 13 | 12.7 | 10.8 | 3 |  |  |  | 0.815 | 0* |  |
| Meat and poultry (EN%) | | women | 40 | 8.9 | 9.5 | 1.5 | 0.320 | -0.2 |  | 40 | 6 | 5.9 | 0.9 | 0.386 | -0.3 |  | 0.115 | -0.4 |  | 77 | 9.5 | 8.9 | 1 | **0.014** | -0.4 |  | 27 | 4.7 | 5.6 | 1.1 | **0.002** | -0.5* |  | **0.002** | -0.3* |  |
|  |  | men | 59 | 10.5 | 6.4 | 0.8 |  |  |  | 16 | 7.6 | 6.7 | 1.7 |  |  |  | 0.052 | -0.2* |  | 57 | 13.8 | 10.6 | 1.4 |  |  |  | 13 | 15.2 | 12.1 | 3.3 |  |  |  | 0.862 | 0* |  |
| Fish (EN%) | | women | 40 | 1.6 | 1.8 | 0.3 | 0.727 | -0.1 |  | 40 | 1.8 | 2.5 | 0.4 | 0.139 | 0.4 |  | 0.614 | 0.1 |  | 77 | 1.5 | 1.8 | 0.2 | 0.783 | 0 |  | 27 | 1.6 | 2.3 | 0.4 | 0.711 | -0.1* |  | 0.679 | 0* |  |
|  |  | men | 59 | 1.7 | 1.7 | 0.2 |  |  |  | 16 | 0.9 | 1 | 0.2 |  |  |  | **0.047** | -0.2* |  | 57 | 1.4 | 1.9 | 0.2 |  |  |  | 13 | 0.8 | 0.7 | 0.2 |  |  |  | 0.609 | -0.1* |  |
| Spreadable fats (EN%) | | women | 40 | 1.9 | 2.1 | 0.3 | **0.026** | -0.4 |  | 37 | 1.9 | 2.4 | 0.4 | 0.605 | 0.2 |  | 0.943 | 0 |  | 75 | 1.8 | 2.6 | 0.3 | 0.167 | -0.2 |  | 27 | 1.3 | 1.4 | 0.3 | 0.570 | -0.2 |  | 0.510 | -0.1* |  |
|  |  | men | 58 | 3.5 | 4.9 | 0.6 |  |  |  | 15 | 1.6 | 1.8 | 0.5 |  |  |  | 0.056 | -0.2* |  | 57 | 2.5 | 2.7 | 0.4 |  |  |  | 13 | 1.6 | 1.7 | 0.5 |  |  |  | 0.377 | -0.1* |  |
| Eggs (EN%) | | women | 39 | 2 | 2.3 | 0.4 | 0.431 | -0.2 |  | 40 | 1.5 | 1.6 | 0.3 | 0.117 | -0.2* |  | 0.299 | -0.2 |  | 77 | 1.6 | 1.9 | 0.2 | 0.370 | -0.2 |  | 27 | 1.3 | 1.6 | 0.3 | 0.887 | -0.2* |  | 0.454 | -0.1* |  |
|  |  | men | 58 | 2.5 | 3.3 | 0.4 |  |  |  | 16 | 2.7 | 2.7 | 0.7 |  |  |  | 0.446 | -0.1* |  | 57 | 1.9 | 2.3 | 0.3 |  |  |  | 13 | 2.5 | 3.7 | 1 |  |  |  | 0.613 | -0.1* |  |
| Sweets (EN%) | | women | 40 | 19.4 | 14.3 | 2.3 | 0.151 | 0.3 |  | 40 | 17.5 | 8.8 | 1.4 | 0.056 | 0.6 |  | 0.468 | -0.2 |  | 77 | 21.8 | 15.4 | 1.8 | **0.002** | 0.5 |  | 27 | 20.5 | 14.3 | 2.8 | 0.122 | 0.5 |  | 0.798 | 0* |  |
|  |  | men | 59 | 15.8 | 10.9 | 1.4 |  |  |  | 16 | 12.5 | 8.3 | 2.1 |  |  |  | 0.365 | -0.1* |  | 57 | 14.3 | 11.6 | 1.5 |  |  |  | 13 | 13.7 | 9 | 2.5 |  |  |  | 0.910 | 0* |  |
| Alcohol (EN%) | | women | 40 | 11.4 | 19.2 | 3 | 0.064 | -0.3 |  | 40 | 19.7 | 24.4 | 3.9 | 0.283 | -0.1* |  | 0.094 | 0.4 |  | 77 | 11 | 18.2 | 2.1 | 0.096 | -0.3 |  | 27 | 16.1 | 26.8 | 5.2 | 0.120 | -0.2* |  | 0.660 | 0* |  |
|  |  | men | 59 | 22.7 | 40 | 5.2 |  |  |  | 16 | 35.6 | 49.7 | 12.4 |  |  |  | 0.072 | -0.2* |  | 57 | 27.1 | 70.2 | 9.3 |  |  |  | 13 | 66.6 | 131.1 | 36.4 |  |  |  | 0.267 | -0.1* |  |
| Water (g/d) | | women | 39 | 2256 | 1781 | 285.2 | 0.178 | -0.3 |  | 40 | 2281 | 1689 | 267.1 | 0.755 | -0.1 |  | 0.949 | 0 |  | 77 | 2823 | 1801 | 205.2 | **0.021** | 0.4 |  | 26 | 2013 | 1586 | 311.1 | 0.663 | 0.2 |  | 0.069 | -0.2* |  |
|  |  | men | 59 | 2757 | 1791 | 233.2 |  |  |  | 14 | 2441 | 1476 | 394.4 |  |  |  | 0.563 | -0.1* |  | 57 | 2098 | 1750 | 231.8 |  |  |  | 11 | 1764 | 1556 | 469.1 |  |  |  | 0.557 | -0.1* |  |

Results of student’s t-test and Mann-Whitney-U test(*) comparison for sex-differences and cohort-specific differences in dietary patterns, dietary choices, and macronutrients in comparison with fatigue.

(*) indicates results of Mann-Whitney-U test.

IBD – Inflammatory Bowel Disease; CC – Control Cohort; sQ-HPF - Screening Questionnaire of Highly Processed Food Consumption; SD – standard deviation; SEM – standard error of the mean..
